# Multiple genetic analyses to investigate the polymorphisms of Chinese Mongolian population with an efficient short tandem repeat panel

**DOI:** 10.3325/cmj.2019.60.191

**Published:** 2019-06

**Authors:** Yating Fang, Tong Xie, Qiong Lan, Xiaoye Jin, Yuxin Guo, Yongsong Zhou, Jiangwei Yan, Bofeng Zhu

**Affiliations:** 1 School of Forensic Medicine, Southern Medical University, Guangzhou, China; 2 Key Laboratory of Shaanxi Province for Craniofacial Precision Medicine Research, College of Stomatology, Xi’an Jiaotong University, Xi’an, China; 3 College of Medicine & Forensics, Xi’an Jiaotong University Health Science Center, Xi’an, China; 4 CAS Key Laboratory of Genome Sciences and Information, Beijing Institute of Genomics, Chinese Academy of Sciences, Beijing, China

## Abstract

**Aim:**

To determine allele frequencies and forensic statistics of 22 autosomal short tandem repeat loci in Chinese Mongolian population.

**Methods:**

Blood specimens were collected from 134 unrelated healthy Mongolian individuals, and 22 short tandem repeat loci were co-amplified and genotyped. Allele frequencies and forensic parameters were calculated, and population genetic differences were analyzed among Mongolian population and other eight Chinese populations: Northern Han, Guangdong Han, Chengdu Han, Xinjiang Hui, Xinjiang Uygur, Hainan Li, Qinghai Tibetan, and Hainan Han.

**Results:**

All the loci were in the Hardy-Weinberg equilibrium, and after Bonferroni correction there was no linkage disequilibrium between them. The allele frequencies of these 22 loci were between 0.0037 and 0.3657. This panel had high discriminating power and genetic polymorphism in the Mongolian population, with combined power of discrimination of 0.999999999999999999999999998399 and combined probability of exclusion of 0.9999999999566925. Structure analysis showed no evidence that these nine Chinese populations had different component distribution. However, genetic distance analysis showed significant differences among them (*P* < 0.05).

**Conclusion:**

The combined application of these 22 loci could be useful for forensic purposes in the Mongolian population. Mongolian population had smaller genetic distances from the populations in northern China (Northern Han, Xinjiang Uygur, and Xinjiang Hui) than from the populations in Hainan province (Hainan Han and Hainan Li populations).

Xinjiang province is an ethnic autonomous region in the northwest of China, bordering Russia, Pakistan, India, Kazakhstan, Tajikistan, Kyrgyzstan, Afghanistan, and Mongolia. Historically, the province was home to an important route on the Silk Road, functioning as China’s gateway to the west. It is inhabited by 47 ethnic groups, including Mongolians, which mainly inhabit the Bayingol Mongol Autonomous Prefecture, Hoboksar Mongol Autonomous County, and Bortala Mongol Autonomous Prefecture. Besides these Chinese regions, Mongolians also inhabit Mongolia and parts of Russia. They are predominantly shamanist and speak a language from the Mongolian group of Altaic family.

A short tandem repeat (STR) is a train of repetitive base sequences on the DNA strand. STR genetic polymorphisms can be analyzed by measuring the exact number of repeating units on the DNA. The novel panel used in this study is a STR genotyping system based on capillary electrophoresis analysis with 5-color fluorescence labeling, which encompasses Amelogenin gene and 22 autosomal STR loci: D1S1656, D2S1338, D3S3045, D4S2366, D5S2500, D6S477, D7S3048, D8S1132, D9S925, D10S1435, D11S2368, D12S391, D13S325, D14S608, D15S659, D16S539, D17S1290, D18S535, D19S253, D20S470, D21S1270, and D22-GATA198B05. The 22 loci are distributed in 22 pairs of autosomes, and their amplified fragments are less than 450 bp.

This panel was validated by a previous study (1), which assessed its sensitivity, accuracy, precision, stability, stutter percentage, peak height ratio, and species specificity. It was used to analyze allelic distribution in Northern Han (2), Southern Han (3), Chengdu Han (4), Hainan Li (5), Xinjiang Hui (6), and Xinjiang Uygur (7). In addition, detailed sequence information of these 22 loci was studied by Phillips et al (8). However, it is unknown whether these 22 STR loci are suitable for forensic application in Xinjiang Mongolian population. Based on the published findings, we hypothesized that the 22 loci had high genetic polymorphisms in Xinjiang Mongolian population and could be applied in this population for individual identification and paternity testing. To test these hypotheses, our study determined the allele frequencies of 22 STRs in Chinese Xinjiang Mongolian population, evaluated the system effectiveness of these 22 loci for individual identification and paternity testing in this population, and compared the findings with other reference populations.

## Materials and methods

### Material

This observational population genetics study was conducted in June 2017 at the Xi’an Jiaotong University. A total of 134 peripheral blood samples of volunteer unrelated healthy Mongolian individuals (90 women and 44 men) were collected from Chinese Xinjiang Uygur Autonomous Region and saved in the form of a paper blood collection card. Individuals were considered eligible if they met the following criteria: (i) there were no blood relationships between them, (ii) they all lived in Xinjiang Uygur Autonomous Region for over three generations, (iii) and there was no migration in their family history. Informed consent for study participation and data presentation was obtained from all volunteers before sampling. The study was approved by the Ethics Committee of the Institute for Xi’an Jiaotong University (Approval No. XJTULAC201, Nov 7, 2013).

### DNA analysis

After the extraction of genomic DNA using the Chelex-100 method (9), Amelogenin gene locus and 22 STR loci were co-amplified using the Microreader 23sp ID kit (Suzhou Microread Genetics, Suzhou, China) on the GeneAmp PCR 9700 thermocycler (Applied Biosystems, Foster City, CA, USA) with 25 μL reaction volume. The amplified products were isolated and detected by capillary electrophoresis using the ABI PRISM 3130XL Genetic Analyzer (Applied Biosystems) with reference to internal lane standard Org500 (including 14 different length fragments: 50, 75, 100, 139, 150, 160, 200, 300, 340, 350, 400, 450, 490, 500 bp). Capillary electrophoresis results were analyzed using GeneMapper ID-X 1.3 software (Applied Biosystems). The 9947A was used as a positive control and DNA-free deionized water as a negative control. Our experiments strictly followed the internal control standards of the laboratory of Southern Medical University (Guangzhou, Guangdong, China).

### Statistical analysis

The Hardy-Weinberg equilibrium (HWE) (10) of the 22 autosomal STR loci was tested by using Modified Powerstats software v. 1.2 (11), which was also used to compute allele frequencies and forensic parameters of each locus, ie, power of discrimination (PD), matching probability (MP), power of exclusion (PE), observed heterozygosity (Ho), and polymorphic information content (PIC). The expected heterozygosity (He) for each locus was calculated by Arlequin software v. 3.5 (12), which was applied to determine whether there was a linkage disequilibrium (LD) (13) between these loci. The combined power of discrimination (CPD) and combined probability of exclusion (CPE) were calculated using the respective formulas: CPD = 1–(1–DP_1_)(1–DP_2_)(1–DP_3_)^…^(1-DP_k_); CPE=1–(1–PE_1_) (1–PE_2_) (1–PE_3_)^…^(1–PE_k_). In these two formulas, indicates the number of loci. The allele frequencies of 22 loci were compared between the Mongolian and other reference populations by Arlequin software v. 3.5 (12). The STRUCTURE analysis was performed by using the STRUCTURE software v. 2.3.4 (14). Genetic distances (D_A_) and fixation index (Fst) values in Mongolian and other populations were calculated with DISPAN (15) and Genepop software v. 4.0 (16), respectively. Heat maps were drawn by R software v. 3.4.3 (17) based on the D_A_ and Fst values, and phylogeny trees were drawn by MEGA software v. 6.0 (18) and Phylip software v. 3.69 (19-21). Principal components analysis (PCA) was performed by using and MVSP software v. 3.1 (22). All the used software is freely available.

## Results

### Hardy-Weinberg equilibrium and linkage disequilibrium tests

The *P* values of 22 loci were all greater than 0.05, which meant that none of these loci deviated from the HWE (Table 1). When the loci were tested for linkage disequilibrium, the *P* values of 22 out of 231 pairwise loci were less than 0.05. After applying the Bonferroni correction (23), the adjusted significance level was 0.0002 (0.05/231), which indicated that there was no linkage disequilibrium between these loci (Supplementary Table 1(supplementary Table 1)). In other words, these loci were independent from each other.

**Table 1:** The allele frequencies and forensic parameters for 22 autosomal short tandem repeat loci in Xinjiang Mongolian population (n = 134)*

Alleles	D1S1656	D2S1338	D3S3045	D4S2366	D5S2500	D6S477	D7S3048	D8S1132	D9S925	D10S1435	D11S2368	D12S391	D13S325	D14S608	D15S659	D16S539	D17S1290	D18S535	D19S253	D20S470	D21S1270	D22-GATA198B05
6														0.0410						0.0224		
7										0.0075				0.1754					0.1679			
8				0.0037						0.0187				0.0149		0.0149			0.0112	0.0037		
9			0.2761	0.2948	0.0037									0.1194		0.3022		0.1940	0.0075	0.0187		
9.2						0.0037																
10	0.0037		0.0485	0.0746	0.0373	0.0149				0.0336				0.1978	0.0187	0.1045	0.0224	0.0373	0.0112	0.1231	0.3022	
10.2						0.0112																
11	0.0448		0.0485	0.2537	0.2910	0.0075			0.0112	0.1381				0.2201	0.1269	0.1903	0.0448	0.0224	0.0858	0.0373	0.1007	
11.2						0.0037																
12	0.0560		0.1119	0.1903	0.1716	0.0448			0.0075	0.3657				0.1828	0.2239	0.1903	0.0037	0.1157	0.3657	0.0672	0.0597	
12.2						0.0112																
12.3																				0.0037	0.0560	
13	0.1007		0.1978	0.0709	0.0485	0.2463				0.2313				0.0485	0.1157	0.1716		0.2687	0.2463	0.0970	0.1231	
13.3																				0.0037	0.0224	
14	0.0485		0.2239	0.0933	0.0485	0.1679			0.1082	0.1754					0.0597	0.0261	0.0149	0.2687	0.0858	0.1679	0.2351	0.0037
14.3																					0.0224	
15	0.2948	0.0037	0.0858	0.0187	0.2799	0.2276		0.0037	0.2761	0.0299	0.0037	0.0149			0.1940		0.2052	0.0821	0.0187	0.1940	0.0709	
15.3	0.0112																					
16	0.2612	0.0075	0.0075		0.1007	0.2052		0.0075	0.3022		0.0149		0.0149		0.1642		0.3582	0.0112		0.1381	0.0075	0.0597
16.3	0.0224								0.0037													
17	0.0672	0.0821			0.0187	0.0373	0.0075	0.0821	0.1866		0.1791	0.1231	0.0373		0.0746		0.1493			0.0896		0.1157
17.3	0.0560								0.0037			0.0112										
18		0.1045				0.0075	0.1306	0.2090	0.0933		0.1157	0.2500	0.0448		0.0224		0.1418			0.0149		0.1007
18.3	0.0187																					
19	0.0037	0.1791				0.0075	0.0858	0.1530	0.0075		0.1455	0.1530	0.2425				0.0522			0.0149		0.1045
19.3	0.0075																					
20		0.1045				0.0037	0.1231	0.0933			0.1381	0.1828	0.2724				0.0037			0.0037		0.1007
20.3	0.0037																					
21		0.0187					0.1082	0.1343			0.2463	0.0933	0.2052									0.2761
22		0.0522					0.0970	0.1269			0.1045	0.1007	0.1194				0.0037					0.1642
23		0.1455					0.1418	0.1530			0.0373	0.0597	0.0373									0.0634
24		0.1604					0.1791	0.0336			0.0112	0.0112	0.0261									0.0112
25		0.1045					0.1157	0.0037			0.0037											
26		0.0224					0.0037															
27		0.0075					0.0075															
28		0.0075																				
																						
MP	0.0564	0.0330	0.0635	0.0792	0.0740	0.0661	0.0342	0.0441	0.0828	0.0972	0.0503	0.0478	0.0716	0.0626	0.0481	0.0783	0.0933	0.0764	0.1016	0.0363	0.0581	0.0468
PD	0.9436	0.9670	0.9365	0.9208	0.9260	0.9339	0.9658	0.9559	0.9172	0.9028	0.9497	0.9522	0.9284	0.9374	0.9519	0.9217	0.9067	0.9236	0.8984	0.9637	0.9419	0.9532
PIC	0.7980	0.8654	0.7838	0.7631	0.7615	0.7872	0.8600	0.8425	0.7427	0.7252	0.8215	0.8238	0.7778	0.8069	0.8272	0.7648	0.7532	0.7663	0.7279	0.8627	0.7930	0.8274
PE	0.6527	0.8168	0.6817	0.5963	0.5555	0.6384	0.7260	0.8015	0.5422	0.5034	0.7111	0.6963	0.5291	0.7409	0.5963	0.5689	0.5555	0.6242	0.5689	0.8321	0.6102	0.6963
Ho	0.8284	0.9104	0.8433	0.7985	0.7761	0.8209	0.8657	0.9030	0.7687	0.7463	0.8582	0.8507	0.7612	0.8731	0.7985	0.7836	0.7761	0.8134	0.7836	0.9179	0.8060	0.8507
He	0.8216	0.8810	0.8129	0.7958	0.7939	0.8162	0.8769	0.8620	0.7798	0.7634	0.8441	0.8458	0.8079	0.8328	0.8490	0.7979	0.7846	0.7988	0.7649	0.8785	0.8184	0.8476
*P*	0.8380	0.2931	0.3672	0.9386	0.6115	0.8884	0.6929	0.1685	0.7548	0.6413	0.6530	0.8733	0.1697	0.2112	0.1027	0.6794	0.8109	0.6735	0.6103	0.1629	0.7101	0.9204

*MP – matching probability; PD – power of discrimination; PIC – polymorphic information content; PE – power of exclusion; Ho – the observed heterozygosity; He – the expected heterozygosity; *P* – probability values of exact tests for Hardy-Weinberg equilibrium.

### Allele frequencies and forensic statistical parameters

A total of 227 alleles were detected at these 22 loci (Table 1). The highest number of alleles was detected at the D20S470 locus (16 alleles) and the lowest number at the D16S539 locus (7 alleles). The highest allele frequency was 0.3657 at two loci and the lowest was 0.0037 at 12 loci.

**Figure 1:**
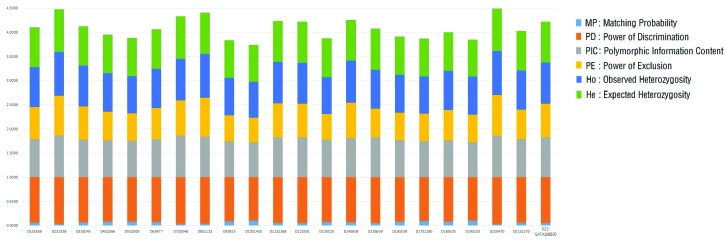
Stacked histogram showing forensic parameters of 22 autosomal short tandem repeat loci in Xinjiang Mongolian population (n = 134).

The MP values ranged between 0.0330 (D2S1338) to 0.1016 (D19S253). In contrast, the PD values of all loci were greater than 0.9, except for D19S253 (0.8984); with a CPD value of 0.999999999999999999999999998399. The Ho values ranged from 0.7463 (D10S1435) to 0.9179 (D20S470) and He values from 0.7634 (D10S1435) to 0.8810 (D2S1338). The heterozygosity of all the loci was greater than 0.7. These results indicate that these loci have high discrimination power in Xinjiang Mongolian population (24,25). The minimum value of PIC was 0.7252 (D10S1435), indicating that all these loci were highly polymorphic. The PE values ranged from 0.5034 (D10S1435) to 0.8321 (D20S470). The CPE value was 0.9999999999566925, which met the Forensic Science DNA Parentage Test Specification issued by the Ministry of Public Security of the People's Republic of China in 2011 (GAT965-2011) that the CPE of the STR panel used in the triad paternity test should not be less than 0.9999 (Table 1) (Figure 1).

### Population genetic analysis

We assessed the differences between Xinjiang Mongolian population and six reference populations in the allele frequencies of these 22 loci by using the analysis of molecular variance (Table 2). There were significant differences at 6, 8, 6, 8, 9, and 17 loci between Mongolian population and Northern Han (Hebei, Henan, Shaanxi) (2), Guangdong Han (3), Chengdu Han (4), Xinjiang Hui (6), Xinjiang Uygur (7), and Hainan Li (5) populations, respectively. In addition, we chose 16 loci as overlapping loci (except for D2S1338, D9S925, D12S391, D16S539, D20S470, and D21S1270) to compare the studied population with the Qinghai Tibetan (26) and Hainan Han (27) populations. Significant differences were observed between Mongolian and Qinghai Tibetan population at 12 loci, and between Mongolian and Hainan Han population at 3 loci.

**Table 2:** The *P* values of the locus-by-locus comparisons based on the allele frequencies in Xinjiang Mongolian and other populations

Loci	Northern Han	Guangdong Han	Chengdu Han	Xinjiang Hui	Xinjiang Uygur	Hainan Li	Hainan Han	Qinghai Tibetan
**D1S1656**	0.6686	0.0968	0.0772	0.8338	0.0029	<0.001	<0.001	0.4330
**D2S1338**	0.0186	0.0264	0.5024	0.0323	0.4565	0.1525	—	—
**D3S3045**	0.1173	0.0362	0.0723	0.2092	0.0479	<0.001	0.0010	0.8397
**D4S2366**	0.0039	0.1232	0.0127	0.0029	0.1652	<0.001	0.0029	0.0010
**D5S2500**	0.5171	0.3118	0.8993	0.5699	0.0968	0.4780	0.1476	0.6256
**D6S477**	0.0978	0.0968	0.1867	0.0254	0.0401	<0.001	0.0244	0.1613
**D7S3048**	0.0821	0.3324	0.0313	0.1496	0.0557	<0.001	<0.001	0.0039
**D8S1132**	<0.001	0.0303	0.0166	0.0039	0.3099	<0.001	<0.001	0.0010
**D9S925**	0.0538	0.0147	0.4291	0.2141	0.0694	0.1417	—	—
**D10S1435**	0.4702	0.4555	0.5816	0.4330	0.1271	0.5562	0.9394	0.5533
**D11S2368**	0.1916	0.0616	0.5748	0.0420	0.0372	0.0274	0.1369	0.1623
**D12S391**	0.2258	0.0127	0.1193	0.5142	0.0635	0.0098	—	—
**D13S325**	0.6061	0.5435	0.7380	0.6334	0.4311	0.0049	0.1281	0.4096
**D14S608**	0.0284	0.0156	0.0068	0.0205	<0.001	0.0020	<0.001	0.1144
**D15S659**	0.7185	0.0782	0.1183	0.6501	0.2483	<0.001	0.0156	0.2434
**D16S539**	0.0362	0.1017	0.0117	0.0098	0.0010	<0.001	—	—
**D17S1290**	0.1271	0.0362	0.0313	0.3558	0.2708	<0.001	0.0010	0.1408
**D18S535**	0.7322	0.0411	0.8974	0.7175	0.0899	<0.001	<0.001	0.2718
**D19S253**	0.0215	0.1720	0.3157	0.1584	0.3529	0.0606	0.0020	0.6843
**D20S470**	0.0938	0.0547	0.3783	0.0156	0.0284	0.0039	—	—
**D21S1270**	0.1105	0.0880	0.3969	0.2297	0.0020	0.0108	—	—
**D22-GATA198B05**	0.1173	0.2669	0.4282	0.2835	0.0274	<0.001	0.0469	0.1593

**Figure 2:**
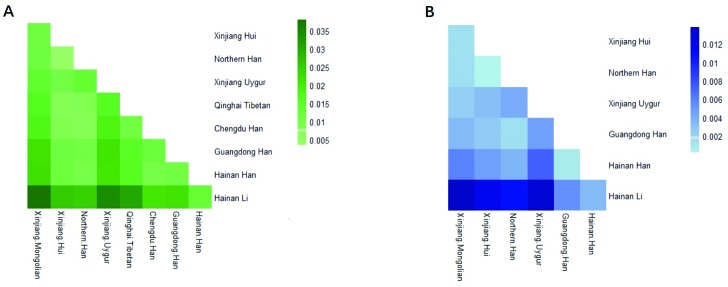
Heat map (**A**) showing the pairwise genetic distances between Xinjiang Mongolian and eight other populations based on allele frequencies of 16 overlapping loci. Heat map (**B**) showing pairwise fixation index (Fst) values of Xinjiang Mongolian and six other populations based on the data of 16 overlapping loci.

The structure analysis of seven populations (Xinjiang Mongolian, Northern Han, Guangdong Han, Chengdu Han, Xinjiang Hui, Xinjiang Uygur, and Hainan Li) offered no evidence that they had different component distribution. Next, we used a series of bioinformatics methods to analyze the genetic relationships between the populations. D_A_ between the populations were calculated based on allele frequencies of 16 overlapping loci. Fst values (a measure of genetic differentiation) between any two of seven populations (except for Chengdu Han and Qinghai Tibetan) were obtained to quantify the genetic relationships between different groups. D_A_ values ranged from 0.0035 to 0.0379 and Fst values from 0.0002 to 0.0138 (Table 3 and Table 4). Xinjiang Mongolian population had the smallest genetic distances from Xinjiang Hui (D_A_ = 0.0113, Fst = 0.0014), Northern Han (D_A_ = 0.0115, Fst = 0.0015), and Xinjiang Uygur (D_A_ = 0.0141, Fst = 0.0023), and the greatest genetic distance from Hainan Li (D_A_ = 0.0379, Fst = 0.0138). Overall, the relationships among these populations were relatively close (Figure 2).

**Table 3:** The pairwise genetic distance values based on the allele frequencies of 16 loci in Xinjiang Mongolian and eight reference populations

Populations	Xinjiang Mongolian	Xinjiang Hui	Northern Han	Xinjiang Uygur	Qinghai Tibetan	Chengdu Han	Guangdong Han	Hainan Han
**Xinjiang Hui**	0.0113							
**Northern Han**	0.0115	0.0035						
**Xinjiang Uygur**	0.0141	0.0105	0.0134					
**Qinghai Tibetan**	0.0163	0.0067	0.0073	0.0160				
**Chengdu Han**	0.0175	0.0077	0.0067	0.0180	0.0107			
**Guangdong Han**	0.0212	0.0116	0.0105	0.0208	0.0155	0.0121		
**Hainan Han**	0.0217	0.0117	0.0096	0.0206	0.0160	0.0099	0.0108	
**Hainan Li**	0.0379	0.0253	0.0242	0.0346	0.0305	0.0205	0.0214	0.0135

**Table 4:** The pairwise fixation index values based on allele frequencies of 16 loci in Xinjiang Mongolian and six reference populations

Populations	Xinjiang Mongolian	Xinjiang Hui	Northern Han	Xinjiang Uygur	Guangdong Han	Hainan Han
**Xinjiang Hui**	0.0014					
**Northern Han**	0.0015	0.0002				
**Xinjiang Uygur**	0.0023	0.0031	0.004			
**Guangdong Han**	0.0033	0.0025	0.0015	0.0046		
**Hainan Han**	0.0059	0.0046	0.0036	0.0072	0.0008	
**Hainan Li**	0.0138	0.012	0.0109	0.0133	0.0053	0.0033

**Figure 3:**
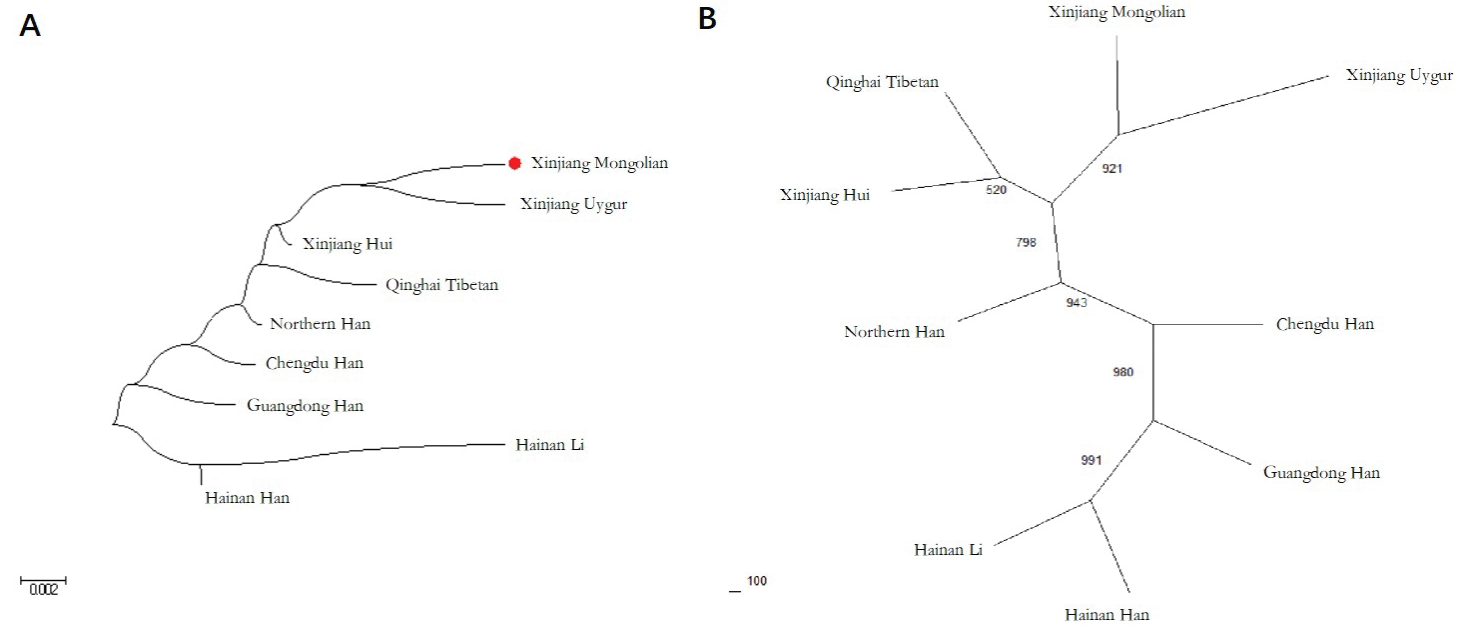
Phylogenetic tree (**A**) showing the relationships between Xinjiang Mongolian and eight other populations based on the results of genetic distance analysis. Phylogenetic tree (**B**) showing the relationships between Xinjiang Mongolian and eight other populations based on the allele frequencies of 16 overlapping loci.

**Figure 4:**
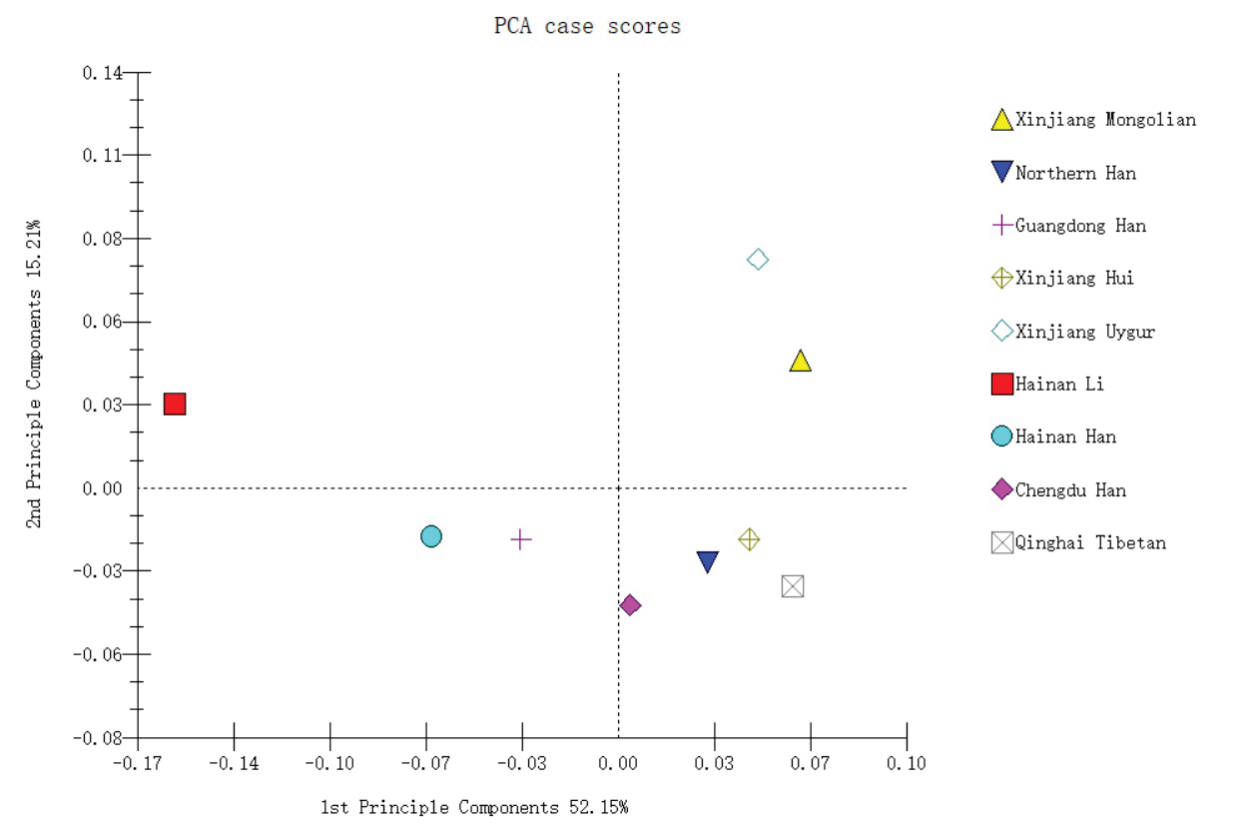
Principal component analysis based on the allele frequencies of 16 short tandem repeat loci of Xinjiang Mongolian and eight other populations.

Based on D_A_ values and allele frequencies we constructed two phylogenetic trees (Figure 3). The populations were divided into two sub-branches. Hainan Han and Hainan Li formed the first sub-branch and other populations formed the second one. In the second sub-branch, Xinjiang Mongolian and Xinjiang Uygur clustered together, followed by Qinghai Tibetan and Xinjiang Hui, and then clustered with the Han populations from different regions. PCA analysis also showed the aggregation of populations. Xinjiang Mongolian and Xinjiang Uygur gathered in the upper right corner, while Xinjiang Hui, Qinghai Tibetan, Northern Han, and Chengdu Han gathered in the lower right corner. In the lower left corner there were Guangdong Han and Hainan Han, while Hainan Li was far away from other populations (Figure 4). The results indicate that the genetic distances between Xinjiang Mongolian and populations in the northern regions of China (Northern Han, Xinjiang Uygur, and Xinjiang Hui) were even smaller. On the other hand, the distances from Hainan Li and Hainan Han populations were large. This is consistent with the previous results (28).

## Discussion

Xinjiang Mongolians had high polymorphism at these 22 STR loci, which confirmed that the combined application of these loci was appropriate for individual identification and paternity testing in this population. In conclusion, our hypotheses were confirmed. Population genetic analysis revealed the genetic relationships between Xinjiang Mongolian and other eight Chinese populations.

With the advancement of science and technology, several new technologies and genetic markers, like next-generation sequencing and single nucleotide polymorphisms, have become widely used. However, due to the lack of databases for new genetic markers, STR typing is still used in the forensic practice. The Federal Bureau of Investigation laboratory in 1997 selected 13 autosomal STRs as core loci of Combined DNA Index System (CODIS) (29), which was in 2017 expanded to 20 STRs (30). Commercially most available STR kits are based on these core loci (31-33). In recent years, these core loci have been complemented by more and more new non-CODIS loci to gain additional genetic information and further improve the discriminatory power (8). Among the studied 22 loci, only four were CODIS loci (D1S1656, D2S1338, D12S391, and D16S539) (30), which increased DNA marker coverage in forensic application. Among other 18 loci, as far as we know, D9S925, D20S470, and D21S1270 are new loci adopted only by this system, which are not included in other commercial kits (8). In fact, newly-adopted STRs should be cautiously used. To verify whether new STRs are suitable for forensic application, it is necessary to perform their detailed genomic characterization and conduct a number of population surveys (8). Detailed studies of the gene sequence information of these 22 loci, especially the newly adopted non-CODIS STRs, as well as the validation studies on the sensitivity, accuracy, and species specificity of this new panel have been performed (1,8). Polymorphisms of these loci in Han, Li, Hui, and Uygur populations in some regions of China have also been reported (2-7). On the basis of these studies, we analyzed the genetic polymorphism of these loci in Mongolian population in Xinjiang.

The sample size in this study (n = 134) was based on previous studies (34,35). Given that the HWE tests were the basis of the population genetics study, the *post hoc* power analysis of HWE tests was performed by R version 3.6.0 (36) (Supplementary Table 2(supplementary Table 2)). The results showed that 17 out of 22 loci had power greater than 0.8. Although *post hoc* power analysis has some limitations in sample size evaluation (37), it indicated that in future studies we may need to increase the sample size to obtain more genetic polymorphism information about the other five loci (especially D3S3045 and D9S925 with power less than 0.5).

Our study confirmed the forensic applicability of these 22 loci in Xinjiang Mongolian population. However, due to the small number of population data on this new system (currently only eight populations have available data), the genetic relationships have to be interpreted in light of certain limitations. In order to further conduct population research and explain the origin of Mongolians, the genetic characteristics of these 22 loci should be evaluated in other populations and genetic characteristics of Mongolians at other loci should be assessed.
