# Supplementary material for: Multiple genetic analyses to investigate the polymorphisms of Chinese Mongolian population with an efficient short tandem repeat panel
Source: Croat Med J. 2019 Jun;60(3):191–200. doi: 10.3325/cmj.2019.60.191 (PMC6563180; doi:10.3325/cmj.2019.60.191)
Supplement: Supplementary Table 1 [file CroatMedJ_60_s006.pdf]

Supplementary Table 1. The *P* values of linkage disequilibrium at 22 short tandem repeat loci in Xinjiang Mongolian population.

Loci	D1S1656	D2S1338	D3S3045	D4S2366	D5S2500	D6S477	D7S3048	D8S1132	D9S925	D10S1435	D11S2368	D12S391	D13S325	D14S608	D15S659	D16S539	D17S1290	D18S535	D19S253	D20S470	D21S1270
D2S1338	0.2253	-	-	-	-	-	-	-	-	-	-	-	-	-	-	-	-	-	-	-	-
D3S3045	0.2129	0.6761	-	-	-	-	-	-	-	-	-	-	-	-	-	-	-	-	-	-	-
D4S2366	0.2859	0.7263	0.7443	-	-	-	-	-	-	-	-	-	-	-	-	-	-	-	-	-	-
D5S2500	0.5107	0.9386	0.7508	0.3791	-	-	-	-	-	-	-	-	-	-	-	-	-	-	-	-	-
D6S477	0.7768	0.0362	0.0631	0.4180	0.6498	-	-	-	-	-	-	-	-	-	-	-	-	-	-	-	-
D7S3048	0.5967	0.9549	0.8638	0.4900	0.6134	0.8863	-	-	-	-	-	-	-	-	-	-	-	-	-	-	-
D8S1132	0.8452	0.7895	0.4667	0.7310	0.3145	0.1291	0.3655	-	-	-	-	-	-	-	-	-	-	-	-	-	-
D9S925	0.4518	0.0237	0.4003	0.3828	0.6824	0.0810	0.2203	0.1794	-	-	-	-	-	-	-	-	-	-	-	-	-
D10S1435	0.6888	0.8724	0.7316	0.9983	0.8361	0.1595	0.2991	0.5305	0.4507	-	-	-	-	-	-	-	-	-	-	-	-
D11S2368	0.6056	0.9746	0.5774	0.8855	0.0107	0.9790	0.2008	0.1596	0.7689	0.0360	-	-	-	-	-	-	-	-	-	-	-
D12S391	0.0130	0.6817	0.4868	0.0963	0.5930	0.0271	0.3458	0.1392	0.3761	0.1821	0.2750	-	-	-	-	-	-	-	-	-	-
D13S325	0.7837	0.1910	0.4656	0.2900	0.1255	0.0026	0.2243	0.8612	0.8099	0.4300	0.2102	0.2596	-	-	-	-	-	-	-	-	-
D14S608	0.5768	0.3689	0.1221	0.8755	0.2370	0.4686	0.7125	0.2473	0.0486	0.4097	0.0328	0.2382	0.4683	-	-	-	-	-	-	-	-
D15S659	0.4564	0.0335	0.1316	0.5005	0.7457	0.7945	0.0026	0.4712	0.7709	0.5082	0.5239	0.7074	0.3545	0.9417	-	-	-	-	-	-	-
D16S539	0.0426	0.8503	0.4342	0.3520	0.8676	0.1565	0.0686	0.5047	0.2312	0.3547	0.1607	0.0669	0.2026	0.0270	0.6662	-	-	-	-	-	-
D17S1290	0.9117	0.1805	0.5959	0.5604	0.4000	0.7736	0.0210	0.2717	0.6311	0.5060	0.4248	0.3661	0.6166	0.6123	0.0655	0.7089	-	-	-	-	-
D18S535	0.9646	0.3275	0.5594	0.1557	0.3331	0.1226	0.9371	0.3631	0.8639	0.7437	0.2341	0.3704	0.9459	0.6534	0.1230	0.4815	0.3697	-	-	-	-
D19S253	0.3798	0.1678	0.3637	0.9744	0.0582	0.3018	0.4636	0.2382	0.6223	0.0255	0.5510	0.1347	0.0408	0.1087	0.5586	0.3314	0.1167	0.2047	-	-	-
D20S470	0.4813	0.9552	0.0148	0.0627	0.6505	0.0359	0.4976	0.8157	0.5407	0.2684	0.1599	0.3494	0.6352	0.6518	0.2463	0.6217	0.8966	0.1819	0.4457	-	-
D21S1270	0.1669	0.6448	0.3382	0.1848	0.1509	0.4760	0.5768	0.1709	0.3080	0.5187	0.6253	0.3095	0.0268	0.0331	0.2225	0.2526	0.7466	0.4372	0.7319	0.4054	-
D22-GATA198B05	0.4271	0.2936	0.0156	0.4665	0.7908	0.7381	0.2626	0.3158	0.6629	0.5395	0.0981	0.3412	0.0668	0.5338	0.9821	0.4485	0.6009	0.6200	0.7206	0.4304	0.0158
