# Supplementary material for: Multiple genetic analyses to investigate the polymorphisms of Chinese Mongolian population with an efficient short tandem repeat panel
Source: Croat Med J. 2019 Jun;60(3):191–200. doi: 10.3325/cmj.2019.60.191 (PMC6563180; doi:10.3325/cmj.2019.60.191)
Supplement: Supplementary Table 2 [file CroatMedJ_60_s007.pdf]

Supplementary Table 2. The statistical powers of Hardy-Weinberg equilibrium test at 22 short tandem repeat loci in Xinjiang Mongolian population. (n=134)

loci	statistical powers
D1S1656	0.9724846
D2S1338	0.9988094
D3S3045	0.3283840
D4S2366	0.8635657
D5S2500	0.8906640
D6S477	0.9998919
D7S3048	0.9456977
D8S1132	0.9655059
D9S925	0.4746404
D10S1435	0.9352855
D11S2368	0.8063220
D12S391	0.8251311
D13S325	0.7956579
D14S608	0.8118105
D15S659	0.9764923
D16S539	0.6272216
D17S1290	0.9999997
D18S535	0.8558351
D19S253	0.9856654
D20S470	0.9997840

D21S1270	0.5635982
D22-GATA198B05	0.9529631
